# Transdermal Diagnosis of Malaria Using Vapor Nanobubbles

**DOI:** 10.3201/eid2107.150089

**Published:** 2015-07

**Authors:** Ekaterina Lukianova-Hleb, Sarah Bezek, Reka Szigeti, Alexander Khodarev, Thomas Kelley, Andrew Hurrell, Michail Berba, Nirbhay Kumar, Umberto D’Alessandro, Dmitri Lapotko

**Affiliations:** Rice University, Houston, Texas, USA (E. Lukianova-Hleb, D. Lapotko);; Baylor College of Medicine, Houston (S. Bezek, R. Szigeti);; Ben Taub General Hospital, Houston (S. Bezek, R. Szigeti);; X Instruments LLC, Fremont, California, USA (A. Khodarev);; Precision Acoustics Ltd, Dorset, UK (T. Kelley, A. Hurrell); Standa UAB, Vilnius, Lithuania (M. Berba);; Tulane University, New Orleans, Louisiana, USA (N. Kumar);; Medical Research Council, Banjul, The Gambia (U. D’Alessandro);; London School of Hygiene and Tropical Medicine, London, UK (U. D’Alessandro)

**Keywords:** malaria, bloodless, hemozoin, nanobubble, laser, noninvasive, parasites, vector-borne infections, diagnosis, *Anopheles* spp., *Plasmodium falciparum*

## Abstract

A fast, precise, noninvasive, high-throughput, and simple approach for detecting malaria in humans and mosquitoes is not possible with current techniques that depend on blood sampling, reagents, facilities, tedious procedures, and trained personnel. We designed a device for rapid (20-second) noninvasive diagnosis of *Plasmodium falciparum* infection in a malaria patient without drawing blood or using any reagent. This method uses transdermal optical excitation and acoustic detection of vapor nanobubbles around intraparasite hemozoin. The same device also identified individual malaria parasite–infected *Anopheles* mosquitoes in a few seconds and can be realized as a low-cost universal tool for clinical and field diagnoses.

## Abstract 2

Our laser device rapidly and noninvasively detected malaria in a patient and identified parasite-positive mosquitoes.

Malaria control and elimination would benefit greatly from an efficient and universal diagnostic tool that is fast (provides results in seconds), noninvasive and safe (uses no blood sampling or reagents), simple to use (can be operated by nonmedical personnel), sensitive and specific (detects low-level asymptomatic infections), and inexpensive and that detects malarial infection in humans and in mosquitoes (*1*–*21*). We recently proposed a transdermal blood- and reagent-free approach based on hemozoin-generated vapor nanobubbles (H-VNBs) (*22*) in which malaria parasite–specific endogenous nanocrystals of hemozoin are optically excited in vivo with a safe and short laser pulse (delivered to blood vessels through the skin). The light is converted into nonstationary localized heat that evaporates the adjacent nanovolume of liquid and thus generates an expanding and collapsing vapor nanobubble inside the parasite. The nanosize and high optical absorbance of hemozoin provide higher malaria infection specificity of these H-VNBs than does any normal blood and tissue components (*23*–*26*). Their transient expansion and collapse result in a noninvasive pressure pulse that is easily detected through the skin with an ultrasound sensor. In our preliminary studies (*22*), H-VNBs detected parasitemia as low as 0.0001% in vitro (human blood), and 0.00034% in vivo (transdermal detection in animals), with no false-positive signals. Therefore, H-VNB might be able to detect extremely low parasite densities provided the method can be applied to humans or mosquitoes simply and inexpensively.

To determine the technical and medical feasibility of H-VNBs for malaria diagnosis and screening, we prototyped a diagnostic device and evaluated it in a patient with confirmed malaria and in noninfected persons as controls. We also evaluated the device in *Plasmodium falciparum*–infected mosquitoes.

## Materials and Methods

### Prototype Design and Algorithms

**Figure 1:**
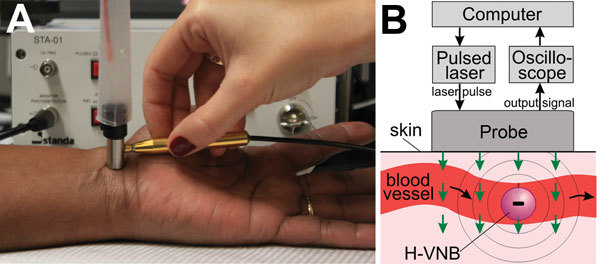
A) Experimental laboratory prototype of a malaria diagnostic device with the pulsed laser and the integrated probe shown being scanned across a human wrist. B) Functional diagram of the prototype and the principle of transdermal optical excitation and acoustic detection of vapor nanobubbles around hemozoin in malaria-infected cells exposed to the laser pulses (green arrows). H-VNB, hemozoin-generated vapor nanobubble.

The laboratory prototype (Figure 1, panel A) comprised the newly designed compact low-cost pulsed laser (532 nm, 10 μJ, 200 ps; Standa, Vilnius, Lithuania). The laser pulse is delivered to the skin by the combination probe at the fluence 36 mJ/cm^2^ (Figure 1, panel B). The probe was developed for transdermal diagnostics and includes an optical fiber guide and a custom acoustic sensor with a preamplifier that is integrated in 1 compact hand-held unit. In response to each laser pulse, the probe detects an acoustic pulse and generates an output electrical signal as an acoustic trace (Figure 1). Its output signals were collected and analyzed with custom-designed software (NI LabVIEW, Austin, TX, USA) by using a signal amplifier, digital oscilloscope (LeCroy 42X; Teledyne LeCroy, NY, USA), and computer. The peak-to-peak amplitude *A* of the acoustic trace obtained in response to each laser pulse was measured and presented as a histogram for 400 sequential laser pulses. A malarial infection–negative trace histogram was used to determine “the malaria threshold” *T* as the maximum amplitude for the malarial infection–negative signal. Any trace with an amplitude above that threshold was considered to be hemozoin (malarial infection)–positive. To quantify the infection, we counted the incidence rate of malarial infection–positive traces *IR* (the probability of the trace incidence with an amplitude above the malaria threshold calculated for 400 laser pulses) and calculated the Hemozoin Index (HI) (*22*): HI = IR(A – T).

### Monitoring of Transient Vapor Nanobubbles

**Figure 2:**
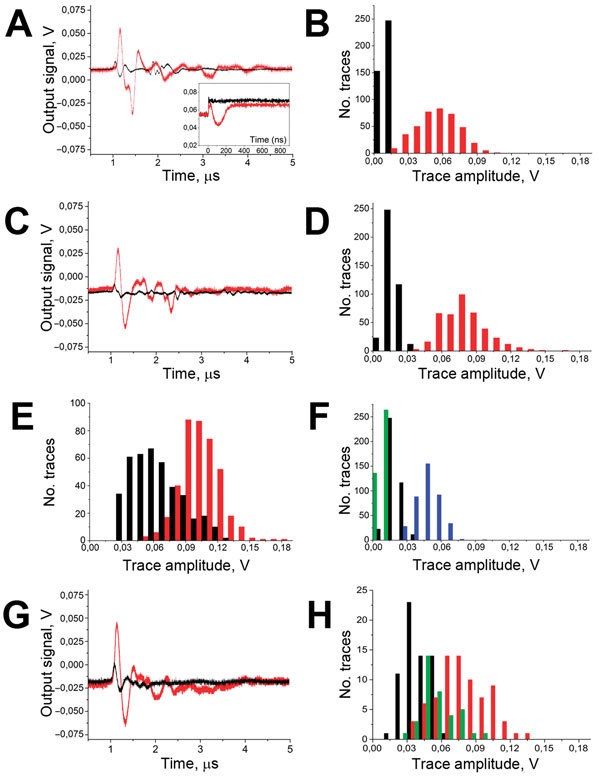
A) Acoustic traces obtained in vitro in response to a single laser pulse exposure (532 nm, 36 mJ/cm^2^ delivered at 0 time point) from the sample of whole human blood (black) and for the same blood sample after the addition of hemozoin in the concentration corresponding to the parasitemia level of 0.8% (red). y-axis shows the output signal of the acoustic sensor, Inset: optical scattering time-responses obtained for these 2 samples show the transient bulk heating of the blood (black) and the generation of transient vapor nanobubble (red). B) Histograms of the trace amplitudes for 400 traces obtained under identical conditions for the above 2 samples. y-axis indicates number of traces with the amplitude in the specific range. C) Acoustic traces obtained from the wrist veins of an uninfected volunteer with light dark skin (black) and from a malaria-diagnosed patient with light dark skin (red). D) Histograms of the acoustic trace amplitudes obtained from the wrist veins of an uninfected volunteer with light dark skin (black) and for a malaria-diagnosed patient with similar light dark skin (red). E) Histograms of the acoustic trace amplitudes obtained from the earlobes of an uninfected volunteer (black) and for a malaria-diagnosed patient (red). F) Histograms of the acoustic trace amplitudes obtained for healthy volunteers with different skin darkness: green, pale light skin; black, light dark skin; blue, dark skin. G) Acoustic traces obtained transcuticle from individual mosquitoes: fed with uninfected blood and oocyst negative (black) and fed with malaria-infected blood and oocyst positive (red). H) Histograms of the acoustic trace amplitudes obtained from oocyst-negative (black) and oocyst-positive (red) mosquitoes and mosquitoes fed with uninfected blood mixed with hemozoin at 60 μg/mL (green).

The direct monitoring of transient vapor nanobubbles in response to a single laser pulse uses our optical scattering method (*27*,*28*). This method used time-resolved optical scattering of a probe continuous laser beam of very low power (633 nm, 0.1 mW). The probe laser beam was focused on the blood sample collinearly with the excitation laser pulse. The axial intensity of the probe laser beam that passed through the blood sample was monitored with a photodetector. The response to the laser pulse included bulk heating and generation of a transient vapor nanobubble. The bulk heating of the exposed blood volume (without generation of a vapor nanobubble) was detected optically by using a thermal lensing effect that revealed the fast heating and gradual cooling of the exposed volume (Figure 2, panel A, black line in inset). The generation of an expanding and collapsing vapor nanobubble created a strong localized scattering of the probe laser beam by the vapor–liquid boundary, and this effect reduced the probe beam intensity with the bubble diameter (Figure 2, panel A, red line in inset). A vapor nanobubble–specific signal typically is shaped like an inverted bell and represents the growth and collapse of the bubble.

### Patient with Confirmed Malaria

The patient was admitted to Ben Taub General Hospital, Harris Health System (Houston, TX, USA), with fever, myalgia, abdominal pain, nausea, and vomiting for the previous 4 days and no history of malaria chemoprophylaxis. Malaria identification and speciation was done by microscopy (thin, Wright-Giemsa–stained, peripheral blood smears) and a rapid malaria antigen test (BinaxNow Malaria; Alere Scarborough, Inc., Scarborough, ME, USA). Both tests confirmed a *P. falciparum* malaria infection. By the time of the H-VNB test, the patient had already received antimalarial drugs (doxycycline, malarone, quinidine, and quinine) for 24 h. During hospitalization, the patient had mild hemolytic anemia and thrombocytopenia, neither of which required transfusion of blood products. In addition to the antimalarial medications and to provide symptomatic relief, the patient received intravenous fluids, antiemetics, and antipyretics.

### Diagnostic Locations

We found wrist and ear lobe veins to be the optimal location for the test. Fingertips were also explored but were inadequate because some persons develop very thick and rigid skin patterns that prevent efficient transdermal delivery of a laser pulse to the blood vessels.

### Influence of Skin Tone

The difference between the amplitudes of the in vitro background traces from intact blood (Figure 2, panel B, black bars) and those in vivo from a malaria-negative volunteer (Figure 2, panel D, black bars) accounts for the additional contribution of the optical absorbance by melanin in dark skin. The increase in the bulk optical absorbance consequently increased the bulk transient heating (without the generation of H-VNBs) and thus increased the average trace amplitude of the background trace from 10.7 ± 1.7 mV in blood alone (in vitro) to 18.1 ± 5.4 mV in blood and skin (in human) as can be seen by comparing the black histograms in Figure 2, panels B and D. We further studied the influence of skin tone on the background trace in 5 healthy volunteers with different skin tones (Figure 2, panel F). For 532-nm wavelength light, the predictable increase in the background trace amplitude resulted from the higher concentration of the skin pigment, melanin, which determines skin darkness (tone). Therefore, in malaria diagnosis, reference malaria-negative data (histogram and threshold) should be linked to the same level of skin tone as in the malaria patient. Although the optical absorbance of skin pigment and hemoglobin is not as high as that for hemozoin (*23*,*24*) and is not sufficient to generate vapor nanobubbles under the laser pulse duration and fluence used (*29*), the bulk transient heat released by hemoglobin and melanin generates a background acoustic trace, which limits the signal-to-noise ratio for the H-VNB method and thus increases the detection threshold for malaria infection. This limitation will be alleviated by developing a “malaria-specific” pulsed laser with a wavelength ≈672 nm—the focus of our ongoing effort because such lasers are not currently available. The optical absorbance of hemoglobin and melanin at 672 nm is much lower than that at 532 nm, and the amplitudes of the background signals and their dependence on skin tone will be reduced. At the same time, the laser pulse energy efficacy of H-VNB generation at 672 nm is similar to that at 532 nm, as we demonstrated previously (*22*).

### Mosquito Model

For infection in mosquitos, female *Anopheles gambiae* mosquitoes were fasted for 6 hours and fed on infected blood by using jacketed membrane feeders warmed to 37°C by a circulating water bath. Briefly, cultured *P. falciparum* (NF54 strain) gametocytes were diluted to 0.3% gametocytemia, 50% normal human erythrocytes and human serum for 15 min. After removing unfed mosquitoes, blood-fed mosquitoes were maintained at 26°C and 70%–80% relative humidity on 10% dextrose. Ten days later, 15 randomly selected mosquitoes were dissected to determine the oocyst numbers, and 2 other groups were used for the device evaluation. Midguts were stained with 0.1% mercurochrome and oocysts counted microscopically. Each of the 15 mosquitoes had 9–151 oocysts (median 50). The remaining mosquitoes were killed by freezing at −20°C before analysis using the prototype device. Mosquitoes fed on uninfected blood and maintained as described were used as negative controls (uninfected and oocyst negative). For further use as positive controls, mosquitoes were fed on uninfected blood containing 60 μg/mL hemozoin.

All procedures were approved by the corresponding internal review board committees at Rice University and Baylor College of Medicine (for Ben Taub Hospital). The patient and the volunteers provided informed consent.

## Results and Discussion

We have prototyped a diagnostic device (Figure 1) and evaluated it in a malaria patient and uninfected controls and in malaria infection–positive mosquitoes. Initial in vitro validation of the designed prototype used samples of whole human blood without (uninfected blood) and with hemozoin (#tlrl-hz; InvivoGen, San Diego, CA, USA) (a proxy for malaria parasite–infected blood [*22*]). The sample cuvette modeled the blood vessel by using a skin-colored film, a channel with blood 1 mm deep, and an acoustically dampening bottom. The bulk optical absorption of the laser pulse at 532 nm by normal whole blood (mainly by hemoglobin) produced the background trace (Figure 2, panel A, black line) associated with the thermo-elastic effect (*30*) (a heat-driven transient pressure rise). However, no vapor nanobubbles were generated because the laser fluence applied was well below the vapor nanobubble generation threshold for any normal blood components (*22*). The absence of vapor nanobubbles was confirmed experimentally by monitoring the laser pulse-exposed sample volume with an optical scattering method (*22*,*27*) by using a low-power probe laser beam at 633 nm and monitoring its time-response to the excitation laser pulse (Figure 2, panel A, black line in inset). In normal blood, the optical scattering time-response to a single laser pulse indicated incremental transient bulk heating without generating a vapor nanobubble. Previously we have shown that such a bulk photothermal effect does not cause any detectable detrimental effects at the molecular and cellular levels (*22*). Adding hemozoin nanocrystals to the blood at a concentration of 23 μg/mL, (which corresponds to ≈0.8% of parasitemia [*9*]), resulted in a completely different acoustic trace under the same excitation and detection conditions (Figure 2, panel A, red line). This trace was attributed to H-VNBs, which were directly detected in the same sample by optical scattering with optical time-responses of 50–100 ns duration and the H-VNB-specific shape, which revealed the vapor bubble expansion and collapse without any recoil (Figure 2, panel A, red line in inset). Unlike the background acoustic traces obtained from the normal blood, the H-VNB acoustic traces yielded 5-fold higher peak-to-peak amplitudes and thus were easily differentiated from the blood background traces in the trace amplitude histograms (Figure 2, panel B).

To obtain proof of the device feasibility, the prototype was further tested in human volunteers who did not have malaria and on a malaria patient with a similar skin tone (dark). We applied the probe to wrists and earlobes and positioned it over subcutaneous vessels. Thus, laser pulses were delivered to blood through the skin. No blood samples were taken, and no reagents were applied. Acoustic traces in response to each of 400 laser pulses (of the same fluence as described earlier) were collected simultaneously with laser irradiation (within 20 seconds total) and analyzed statistically in real time. In the malaria patient, the parasitemia (percentage of infected erythrocytes) was determined by microscopy (thin blood film) and varied from 2% (corresponding to ≈69,000 parasites/μL) 4 hours before the device test to 0.3% (corresponding to ≈8,600 parasites/μL) 9 hours after the device test. As malaria-negative controls, we used healthy volunteers with similar skin tone and under the conditions and procedure applied to the malaria patient. Acoustic traces from the wrist of the malaria patient (Figure 2, panel C, red line) showed the H-VNB–specific pattern similar to that of the hemozoin-positive samples in vitro (Figure 2, panel A, red line) and had much higher amplitudes than those obtained from a healthy volunteer with a similar skin tone (Figure 2, panel C, black line). The amplitudes for the traces from the malaria patient were significantly higher than those from the control, and the 2 histograms barely overlapped (Figure 2, panel D). Therefore, these acoustic traces indicated malarial infection in a clinically ill patient. Similar traces were obtained when the device was applied to the earlobes of the malaria patient and volunteers (3 volunteers with dark skin tone were studied) (Figure 2, panel E). The similarity between the wrist and ear lobe results further validates the successful detection of malarial infection by the H-VNB method. A quantitative analysis used the volunteers’ histograms to determine the malaria threshold amplitude and revealed HI values as 42.4 mV and 1.3 mV in the malaria patient for the wrist and earlobe, respectively.

The safety of H-VNB generation in humans is ensured by the safe level of the laser fluence applied, 36 mJ/cm^2^, which is considered to be skin-safe according to federal regulations (*31*). In addition, no short-term (10 min) or long-term (3–4 d) signs of skin damage or irritation were observed in the study participants. These observations are also in line with our previous observation of no damage to the laser-exposed malaria parasite–negative blood cells (*22*). Therefore, the device and procedure developed appear to be safe, and coupled with their blood- and reagent-free nature, deliver a completely noninvasive diagnosis of malaria in humans.

We further studied the influence of skin tone on the background trace for 5 healthy volunteers with different skin tones (Figure 2, panel F). For 532-nm wavelength light, we observed a predictable increase in the background trace amplitude resulting from the increase in the concentration of melanin.

In the third model, we evaluated the device for the rapid noninvasive transcuticle analysis of individual malaria-infected (oocyst-positive) *Anopheles* mosquitoes. Ten acoustic traces were obtained for each mosquito (7 in each group) by scanning the body across the probe under the same laser pulse fluence as described for the human studies. The negative control group (fed with uninfected human blood, no oocysts) returned acoustic traces similar to those for hemozoin- and malaria parasite–negative human blood (Figure 2, panel G, black line). In the malaria-infected mosquitoes, the trace shape and amplitude (Figure 2, panel G, red line) were similar to those obtained for hemozoin- and malaria parasite–positive traces. The histogram of malaria-infected mosquitoes significantly shifted to the right, compared with the negative control group (Figure 2, panel H). Finally, the positive control group of mosquitoes fed with the blood mixed with hemozoin (60 μg/mL) also showed traces of a high amplitude (Figure 2, panel H, green bars). This experiment demonstrated the feasibility of the rapid noninvasive detection of individual oocyst-carrying mosquitoes. We propose that vapor nanobubbles can be generated around residual hemozoin in developing oocysts or similar dense forms of heme formed by the malaria parasites at this stage (*32*,*33*).

These results provide a proof-of-principle for the H-VNB technology as a unique noninvasive transdermal diagnostic tool for malarial infection in humans and mosquitoes. The next step is to optimize the prototype with a malaria-specific laser operating at 672 nm for a better sensitivity (*22*). Such prototype will be evaluated in large-scale studies in humans in clinical and field settings in malaria-endemic countries. Although the estimated cost of a battery-powered device (size of a shoebox) is US $15,000, a single unit will be able to test ≈200,000 persons each year without any additional cost (e.g., specialized staff, facilities, and diagnostic reagents); thus, the cost of the individual test may be well below that of a rapid diagnostic test. The device may be able to diagnose asymptomatic and low-density infections, undetectable by microscopy and rapid diagnostic tests and could be deployed for mass screening and treatment or at border control points (a major advantage would be the speed at which results will be available). The presence of hemozoin in all blood stages and types of malaria parasites (*25*,*26*) ensures the broad and universal application of our method, even without differentiation of the malaria species. The rapid and simple detection of malaria-infected mosquitoes could provide an easy tool to estimate the transmission intensity, contributing to the efforts of malaria transmission reduction and local elimination.
